# Anaerobic Digestion for Producing Renewable Energy—The Evolution of This Technology in a New Uncertain Scenario

**DOI:** 10.3390/e23020145

**Published:** 2021-01-25

**Authors:** Cristián Arenas Sevillano, Alby Aguilar Pesantes, Elizabeth Peña Carpio, Elia J. Martínez, Xiomar Gómez

**Affiliations:** 1 Chemical and Environmental Bioprocess Engineering Group, Natural Resources Institute (IRENA), Universidad de León, Av. de Portugal 41, 24009 León, Spain; cares@unileon.es (C.A.S.); ejmartr@unileon.es (E.J.M.); 2 Departamento de Química y Física Aplicadas, Campus de Vegazana, University of León, 24071 León, Spain; alby.aguilar@gmail.com; 3 Escuela Superior Politécnica del Litoral (ESPOL), Facultad de Ingeniería en Ciencias de la Tierra, Guayaquil EC090112, Ecuador; gepena@espol.edu.ec

**Keywords:** biogas valorization, lignocellulosic pre-treatment, techno-economic performance, process integration, energy production

## Abstract

Anaerobic digestion is a well-known technology with wide application in the treatment of high-strength organic wastes. The economic feasibility of this type of installation is usually attained thanks to the availability of fiscal incentives. In this review, an analysis of the different factors associated with this biological treatment and a description of alternatives available in literature for increasing performance of the process were provided. The possible integration of this process into a biorefinery as a way for producing energy and chemical products from the conversion of wastes and biomass also analyzed. The future outlook of anaerobic digestion will be closely linked to circular economy principles. Therefore, this technology should be properly integrated into any production system where energy can be recovered from organics. Digestion can play a major role in any transformation process where by-products need further stabilization or it can be the central core of any waste treatment process, modifying the current scheme by a concatenation of several activities with the aim of increasing the efficiency of the conversion. Thus, current plants dedicated to the treatment of wastewaters, animal manures, or food wastes can become specialized centers for producing bio-energy and green chemicals. However, high installation costs, feedstock dispersion and market distortions were recognized as the main parameters negatively affecting these alternatives.

## 1. Introduction

Anaerobic digestion is a well-established technology for treating organic wastes with high water content and that are very prone to biological degradation. This technology has been applied worldwide thanks to its capacity to degrade high loads of organic materials and producing biogas. Wastes should be considered as “renewable resources” that can be used to generate new products [1] instead of outputs without any value. Biogas is the main energetic component derived from digestion, but the process also provides a side-stream product (digestate) that may not be so easily valorized. Proper transformation and stabilization of digestate can make this slurry a valuable organic amendment. Digestate has a high content of humic and fulvic substances and nutrients, making it ideal for agronomic use once its biological stability is improved [2].

The dramatic effect of CO_2_ emissions on the global climate, the rapidly changing price of fuels, and social concerns about the depleted fossil fuel reserves, such as crude oil and natural gas, have increased the interest in producing bioenergy derived from biowastes [3]. Anaerobic digestion is a process capable of providing this bioenergy thanks to methane production via biological transformation of substrates. Digestion technology offers major benefits, providing eco-friendly energy and at the same time addressing the waste management crisis [4].

One of the most extended applications of anaerobic digestion technology is the integration of this process into wastewater treatment plants. Wastewater needs to undergo a series of treatments to meet the local allowable discharge limits. Sewage sludge is generated at the same time that the organic loading of wastewater is reduced. This sludge can be stabilized by anaerobic digestion, but it can also be treated in co-digestion reactors to adjust the balance of nutrients and attain economic feasibility of the global process [5]. Basically, digestion is carried out by microorganisms that stabilize the organic materials by transforming them into complex compounds less prone to uncontrollable degradation. This transformation produces a slurry of black or brownish color with a less offensive odor, which is also known as biosolids, if sewage sludge was the original organic material.

Biogas derived from anaerobic digestion can be valorized for producing thermal and electric energy. Biogas mainly consists of methane, carbon dioxide, and low quantities of trace gases. Its composition depends mainly on the type of substrate, process operating conditions, applied organic loading, hydraulic retention time and digester design [6,7]. Biogas can be transformed into biomethane once carbon dioxide is separated and other contaminants are removed. Eliminating these contaminants is of great relevance and this is particularly true for the removal of H_2_S. This compound may cause serious corrosion problems and give rise to operability issues associated with its oxidation products. In the case of CO_2_, the importance of removing this compound is based on the quality standard of biogas and the type of valorization technology this gas will be destined to [8,9,10].

Enhancing the efficiency of biological processes is of great relevance to increase product yield and process performance. The valorization of organics into energy allows one to reduce the carbon footprint of different waste management options. However, attempts for increasing the efficiency of anaerobic digestion do not always have a successful outcome. There exist several treatments for improving the degradation of organics to facilitate the hydrolysis stage. Thermal hydrolysis is a well-developed technology installed as a pre-treatment unit for enhancing biogas production in several wastewater treatment plants. The first full-scale plant for sludge disintegration through the Cambi process was started in Hamar, Norway, in 1995 [11]. Since then, this company has installed several other plants worldwide [12]. Other commercial technologies include BioThelys™, which is a batch technology just as it is the Cambi^TM^ Process (Cambi, Asker, Norway) [13]. Haarslev is a continuous operating technology [14]. Other thermal hydrolysis processes use heat exchangers to increase temperature such as Exelys™ developed by Veolia Water Technologies (Veoli, Libourne, France) [15]. Turbotec^®^ from DMT Environmental Technology (Tualatin, OR, USA) [16,17], and Lysotherm^®^ from Eliquo Water and Energy BV (Barneveld, The Netherlands) [18] are also other commercial technologies available.

Other pretreatments intended for improving hydrolysis include ultrasonication, available at a commercial scale, whereas other treatment options such as microwave pre-treatment, electrokinetic and high-pressure disruption are mechanical pre-treatments studied mainly under laboratory conditions or using small scale prototypes [19,20,21,22,23]. Other pre-treatment methods include the use of chemicals—acidic, alkali, ozonation—[24,25], and the application of advanced oxidation processes [26,27], and biological options (temperature-phased anaerobic digestion and microbial electrolysis cell) [28,29,30] or combinations on any of the above to increase the effectiveness of solubilization. However, the capacity of recovering the heat of the thermal hydrolysis process makes this technology superior when evaluating the efficiency in energy use. The valorization of biogas by using combined heat and power (CHP) units allows for exhaust gas heat to be recovered in a recovery boiler [31], thus fulfilling the thermal needs of the hydrolysis pre-treatment.

The extended application of anaerobic digestion and the high costs associated with these installations have given rise to extensive research activities to increase the efficiency of the process, enhance biogas production and attain economic feasibility. The evaluation of anaerobic digestion of sewage sludge with different substrates has been widely reported in the literature [32,33,34], and it is still under extensive research. There are plenty of reports evaluating the co-digestion of sewage sludge with solid organic wastes, high strength organic streams and different compounds acting as supplements to favor organic degradation [35,36,37]. Regarding this last subject is the addition of conductive carbon materials—char, graphene, graphite, activated carbon—to the digestion process that has gained recent interest. The addition of these materials favors direct interspecies electron-transfer and increases the degradation of volatile fatty acids and proteins [38,39].

Several factors influence the global performance of a process; therefore, a great variety of key points and interacting relationships need to be evaluated for optimizing efficiency and energy recovery. To approach a decarbonized economy by 2050, efficient bioenergy production is essential—playing a major role will be recycling and reuse—towards a “circular economy”. However, as Valero and Valero [40] stated, absolute circularity in transformation processes does not exist, and this is based on the second thermodynamic law dictating that, in each cycle, some quantity and quality of materials is unavoidably lost. Thus, performing energy balances associated with the conversion of biomass would allow establishing processing routes with minimum energetic constraints. When energy is transferred in the form of heat it has a certain quality; this quality can be lost partly or completely by the heat transfer process. The quality of the transferred energy can best be quantified by the “exergy” concept in which energy is divided into two parts: exergy, also called “available work”, is the maximum theoretical work obtainable from the energy [41]. Thus, exergy analysis reflects the theoretical maximum performance of energy because it is based on the first and the second law of thermodynamics, putting emphasis on energy amount and quality at the same time [42].

Animal manures are usually treated by anaerobic digestion, a residue with high organic content but also with high N concentration, which may inhibit methanogens due to the accumulation of ammonia nitrogen in the reactor. Anaerobic digestion is also widely applied to treating the organic fraction of municipal solid wastes (OFMSW). In this case, the presence of improper materials and relatively high concentrations of heavy metals may add complexity to pre-treatment operations intended to prepare a homogenous feeding slurry. The difficulty associated with the separation of inert materials and the contamination with toxic elements make this digestate not suitable for agronomic use. The application of anaerobic digestion for the conversion of crop wastes and agro-industrial wastes is also a suitable management option. Still, the process needs to confront the seasonal availability of these materials, which is restricted to a short period of the year. The disadvantage of low nitrogen levels, which may cause nutrient deficiencies, and the need to apply long residence time in the reactor should be added.

The addition of a co-substrate allows the adjustment of nutrient balance, improves the stabilization of organic matter, and results cost-effectively because different substrates share the same installations [43,44] with the mixture with higher biogas yields. As a consequence, co-digestion is expected to increase the efficiency of the process between 25 and 400% when it is compared to the degradation of a mono-substrate [45] thanks to the increments in organic loading, enhancement in volatile solid removal and higher biogas productivity.

The present manuscript reviews different relevant factors in digestion performance, addressing particular key points regarding industrial application. This manuscript intends to share a different view of anaerobic digestion, from being considered a conventional degradation process to being understood as a key element in clean energy production. Anaerobic digestion is a highly efficient process that has been extensively studied, but there is still a need to set the focus on specific parameters that are crucial, such as capital investment cost, efficiency of degradation and valorization of digestates. This technology can greatly aid in attaining the decarbonization of the economy and play a major role in the future economy system based on the principles of circular economy. It also intends to emphasize the relevance of developing sustainable processes for energy production within the limits of economic feasibility.

## 2. The Effect of Substrate Composition and Digestion Performance

Carbohydrates are present in all types of substrates and particularly plant-derived biomasses and food industry wastes. Substrates from the food processing industry, catering and residential activities are characterized by a readily degradable fraction, which is easily acidified. In recent years, changes in consumer demand have been observed due to preferences for a healthier lifestyle leading to the development of a new market offering fruit and salad products ready to eat. This affects the characteristic of waste produced and represents new valorization opportunities [46]. Another important change in consumer preference has been caused by the global pandemic due to the strict confinement of the population in many countries. These restrictions led to a sudden change in waste composition and an important decrease in the demand of energy [47]. Once the severe spring confinement period has ended, the attempts for reactivating the economy have been intermittent due to the recurrent appearance of infection spreading. This will probably change in one way or the other consumption trends and therefore the quantity and quality of wastes produced.

Fruit and vegetable wastes have high content of saccharides and disaccharides that are easily degradable by anaerobic microorganisms forming volatile fatty acids (VFAs) as intermediaries. When carbohydrates represent a large proportion of the feeding stream, a suppression of methanogenesis may be experienced with the overloading of the digester due to an initial VFA build-up leading to a decrease in reactor liqueur pH [48]. This VFA imbalance may negatively affect the production rate of biogas. This is particularly true for systems working under a feeding recipe where the loading of the reactor is performed in a cyclic manner during short periods of the day [49]. Thus, the biogas production rate is increased right after the feeding procedure, then proceeds at a slower rate until the next feeding procedure starts again. Lower methane composition may be experienced, or variations in production rate are expected based on the accumulation of VFA caused right after the feeding has taken place. Labatut et al. [50] studied the behavior of different types of substrates and their performance when evaluating biochemical methane potential (BMP) tests reporting on different cumulative methane curves with a particular shape directly related to the degradability of substrates. Thus, when operating a digester, special care should be taken on substrate degradation rate, acidification potential, and punctual loadings of the reactor, rather than setting as fixed operating parameters the organic loading rate and hydraulic retention time.

The high acidification potential of carbohydrate-rich substrates, when treated under anaerobic digestion, has aroused interest in transforming the hydrolysis–acidification stage into a fermentative process for hydrogen recovery. The evolution of hydrogen from fermentation is a ubiquitous phenomenon under anoxic or anaerobic conditions, where the oxidation of the organic matter releases electrons that need to be disposed of to maintain electrical neutrality [51]. In these environments, where no oxygen is available, other compounds act as electron acceptors, and therefore, protons are reduced to molecular hydrogen (H_2_) [52]. Dark fermentation, also known as the fermentative production of hydrogen, resembles anaerobic digestion due to its flexibility in assimilating different substrates, with wastes being also the preferred option when considering large-scale implementation [53,54]. In this case, hydrogen is produced from strict and facultative anaerobes (*Clostridia*, *Micrococci*, *Methanobacteria*, *Enterobacteria*, etc.) [55].

The sequential production of H_2_ and CH_4_ has been proposed as a way to increase the economic feasibility and energy recovery of waste treatment plant [56,57,58]. This configuration is a logical approach since both processes have similar capabilities of treating the same type of substrates. However, a second stage is always necessary when producing hydrogen by this fermentation route because acid intermediaries (short-chain fatty acids) and solvents may accumulate in the fermentation broth in the first stage, needing further treatment to stabilize the organic matter completely. The recovery of hydrogen in the first stage and the production of methane in the second one is a more efficient way to extract energy from organics. The mixture of natural gas supplemented with hydrogen is called hythane and its use in combustion engines presents better performance parameters regarding fuel consumption and emissions [59]. This explains the current interest in obtaining a mixture of hydrogen and methane from biological processes, called bio-hythene. Not only is the biological treatment enhanced, but also the final use of biofuels produced is highly benefited.

Bolzonella et al. [60] studied the fermentation of the organic fraction of municipal solid wastes and reported a biogas yield of 490 L/kg VS from the single-stage digestion. However, these same authors reported a hydrogen yield of 24 L/kg VS and 570 L CH_4_/kg VS when evaluating the two-stage process. Thus, the coupling of the results of these two processes is very interesting in terms of the energy content of the gas stream. Similar results were reported by Fernández et al. [61] using cheese whey as substrate in this case. They obtained an H_2_ yield of 12 L/kg COD (chemical oxygen demand) in the first stage and a yield of 340 L CH_4_/kg COD in the second stage, whereas the digestion of this same substrate resulted in a methane yield of 314 L CH_4_/kg COD. There is an urgent need for overcoming the constraints associated with the hydrogen production process as it is, the stability of the fermentation and alkalinity needs for controlling pH. However, the results seem to indicate great flexibility in treating different substrates and a significant potential for integrating this process with conventional waste treatment systems.

Cellulose is another main component of food wastes and agricultural wastes, but it may also be accompanied by a lignin structure, in this latter case. These materials are usually denoted as lignocellulosic wastes. Cellulose is insoluble, and the initial degradation of this compound occurs exocellularly, either in association with the outer cell envelop layer or extracellularly [62]. The availability of electron acceptors usually limits the anaerobic decomposition. During this process, intermediaries such as alcohol and VFAs are formed, needing a syntrophic interaction between bacteria and other organisms such as methanogens to avoid their accumulation and proceed with further degradation of polymer materials [63]. Anaerobic bacteria are able to degrade cellulose thanks to their unique feature of possessing an extracellular multi-enzyme complex, called cellulosome, which is capable of attaching to the cell envelope and the substrate [64].

Cellulose is a polymer of high molecular weight composed of D-glucopyranose units linked by β-1,4-glycosidic bonds with a repeating unit cellobiose. Its structure is not fully understood, but it is in general considered to have a crystalline and an amorphous structure, with this latter one more susceptible to enzymatic degradation [65]. Bacterial degradation of cellulose has been studied by Yamazawa et al. [66], indicating that the degradation reactions of cellulose induced the metabolic dynamics of the microbial community and produced short-chain components, such as acetic and propionic acid, which are mainly metabolized by clostridial species.

Cellulose compounds and hemicellulose are fully degraded by anaerobic microflora leading to the accumulation of chemically recalcitrant aliphatic molecules [67]. Studies of anaerobic degradation of manures have been carried out by Gómez et al. [68,69] applying spectroscopic techniques (nuclear magnetic resonance, NMR) to evaluate the conversion of organic matter. These authors reported complete degradation of cellulose and demonstrated the accumulation of long-chain aliphatic molecules in digestates. However, hydrolysis of cellulose may be considered a hindering step making necessary the use of pre-treatments [70] due to the long time needed for degrading this compound if compared with that of carbohydrates. Chapleur et al. [71] reported cellulose degradation to be accomplished in about 40–50 days under batch anaerobic tests.

Hemicellulose is the other main component of plant material and, therefore, of crop wastes. The lignified material conforming plant cell wall is a complex structure. Xylans are the predominant constituents of hemicellulose in hardwoods and straw, whereas the largest hemicellulose fraction in softwoods is composed of galactomannans [72]. The composite structure of lignocellulosic biomass and the long time needed for degrading cellulose and hemicellulose make it almost imperative to apply pre-treatments to reduce the complexity of lignocellulosic biomass if microbial degradation is intended for obtaining different valuable products. In the case of anaerobic digestion, the conversion of hemicellulose was reported to be higher than that of cellulose under mesophilic conditions by Ghosh et al. [73], who also reported a much lower efficiency than that for cellulose under the thermophilic regimen. Other results from batch degradation of plant main components showed that the biomethane potential of cellulose was higher than that of hemicellulose, but this latter was characterized by easier hydrolysis than the first one. On the contrary, lignin had proven to be difficult to digest [74], experiencing small changes in its native structure when extended digestion periods were applied, resulting in a simpler structure with fewer functional groups than those of the native lignin [75].

Cellulose is also a suitable substrate for biological hydrogen production, although the fermentative process still encounters several technical constraints. The coupling with anaerobic digestion has proven to be effective and the introduction of a recirculation stream from the methanogenic phase to the hydrogen-producing phase allows the recovery of alkalinity, needed for pH control, and also the recycling of microorganisms to keep the first hydrogen-producing stage active [57,76]. Qi et al. [77,78] have proved the successful performance of the recirculating configuration and the feasibility of treating cellulose using this combined approach by evaluating food wastes and paper waste at different mixture ratios. These authors demonstrated that a second mesophilic stage was necessary for allowing the growth of cellulose-degrading bacteria that need to be continuously returned to the hydrogen-producing reactor.

The hydrothermal process has been proposed as a potential technology for aiding in the transformation of lignocellulosic resources into biofuels through fermentation. Value-added chemicals can be produced using new green-conversion routes thanks to the fractionation of lignocellulosic biomass, thus allowing the exploration of new developments into the biorefinery concept. The hydrolysis of hemicellulose produces oligosaccharides, pentose (xylose and arabinose), hexose (glucose, mannose, and galactose), acids (acetic acid, formic acid, and levulinic acid), and furans (furfural and 5-hydroxymethylfurfural), as well as insoluble humins as by-products under harsh conditions [79].

Second-generation biofuels and biogas production from lignocellulosic material offers great potential due to the abundance of this natural resource. It would aid in attaining the decarbonization of the economy. However, the highly heterogeneous structure and the recalcitrant nature of lignocellulose restrict its use as a substrate in biogas plants [80]. Effects associated with the conditions of the thermal pre-treatment and the production of inhibitory compounds need to be well established prior to considering the introduction of a thermal pre-treatment unit to facilitate biomass hydrolysis. Chapleur et al. [71] have reported negative effects due to the presence of phenol when evaluating digestion batch tests of cellulose.

Recalcitrant compounds, such as furfurals, could also be formed at high temperatures during hydrothermal pretreatment, which may hinder the performance of the subsequent biological process [81]. Another important fact that should also be evaluated is the high energy demand of these pre-treatment units and the high capital investment. On the other hand, the main advantage of this pre-treatment is the decrease attained in digester volume because the process can be ended in much shorter times and the amount of remaining digestate is highly reduced.

## 3. Co-Digestion to Increase Reactor Productivity

The co-digestion process can be seen as a promising way for improving the digestibility of cellulose and hemicellulose, balancing nutrients, and attaining buffering effects when high protein compounds are to be used as co-substrates [82]. Co-digestion may be balanced for optimizing methane production, therefore higher methane yields can be obtained for proper carbohydrates, proteins and cellulose ratios [83]. The fact is that large scale digestion plants have to deal with the near source of resources available in their surroundings all year round. Therefore, flexibility of the processing plant is of great relevance to attaining high biogas yields during the whole operating life given the intrinsic variability of available substrates, and options for balancing and establishing specific feeding recipes may not always be possible.

Tufaner and Avşar [84] reviewed the production of biogas when co-digesting different substrates, indicating that the most important premise for obtaining a significant biogas enhancement and producing high-quality digestate is evidently the use of high-quality feedstock. Some feedstocks may be difficult to digest or unsuitable for mono-digestion because of their unfavorable C/N ratios or high lipid content, thus would benefit from co-digesting with manures that have high protein content. Kitchen wastes are a resource prone to acidification and thus are suitable as a co-substrate when digesting manures. Li et al. [85] reported a 116% increment in methane yield when this co-substrate was added to the digestion of cattle manure. Food wastes and sewage sludge have also been proposed as co-digesting mixtures [86,87]. Another relevant co-substrate widely studied due to its high biogas yield and great capacity for boosting biogas production is crude glycerol derived from the biodiesel production process. Paulista et al. [88] reported a 77% increase when adding glycerol as co-substrate to sewage sludge digestion. However, despite its abundance, the price of crude glycerol in the market is still too high to be considered a feasible option [89].

Table 1 reports different values of biogas yields obtained under batch digestion tests and continuous operation. This table shows a great variability in values, with the lowest ones being explained by inhibitory conditions in the system [90]. Good management and treatment of agricultural and livestock wastes must be of vital importance for any country. Spain has a high potential in this field that should be better exploited considering that this country leads the production and export of fruit and vegetables from the EU-28 [91].

Some works have addressed the possibility of using fruits and vegetables to be co-digested with sewage sludge [92,93] or treating this rich carbohydrate and high-quality waste in a decentralized manner to obtain a digestate of outstanding quality for producing high-value agronomic materials [94,95]. When considering alternatives for the management of fruit and vegetable wastes, it is important to take into account the fact that its production is seasonal and, therefore, a large amount may be generated in short periods, and variations of the type of material are also dependent on consumer preference based on the season of the year. In either case, a flexible approach should consider the design of a waste treatment line capable of absorbing modifications in the incoming substrate. For this reason, it is more interesting to take advantage of synergies that may be established between the treatment of food waste and sewage sludge from wastewater treatment plants (WWTPs) that may exert a buffering effect in the operating dynamic of the reactor.

**Table 1:** Methane yields reported in literature for different substrates.

Organic Substrate	Specific Production Potential (M^3^ CH_4_/Kg VS) ^1^	Reference
Livestock manure		
Pig manure	0.30–0.50	[96,97,98,99,100,101]
Poultry manure	0.03–0.11	[90,96,100,101]
Cattle manure	0.11–0.54	[102,103]
Organic industrial waste		
Slaughterhouse waste	0.20–0.80	[104,105]
Brewery waste	0.3–0.51	[106,107]
Sewage sludge (SS) and co-substrate		
SS	0.22–0.45	[35,108,109]
SS + grease	0.4–0.8	[108,110]
SS + glycerol	0.2–0.4	[111,112]
SS + food wastes	0.4–0.6	[86,113]
Energy crops		
Corn stover	0.30–0.40	[97,114]
Sunflower	0.20–0.40	[115,116]
Rapeseed	0.25	[97]
Wheat straw (steam explosion pre-treatment)	0.25–0.35	[117,118]
Rice straw	0.26	[119]
Grass: Napier grass, Canary grass, King grass	0.15–0.60	[120,121,122]
Microalgae and cyanobacteria biomass		
Microalgae *Chlorella* sp.	0.23–026	[99,123]
Microalgae *Nannochloropsis oculata*	0.3–0.35	[124]
Manure + *Arthrospira platensis*	0.48	[125]

^1^ VS: volatile solids.

Proteins are abundant in all organic substrates but mostly in animal-derived wastes. Slaughterhouse waste, pig and chicken manure are residues that have a high content in this material. When treating slaughterhouse waste, ammonia accumulation in the reactor may become an issue if a balancing carbon source is not available in enough quantities. Another residue that should also be considered as good co-substrates is animal carcasses. The treatment of this material is under stringent regulations, but the risk associated with the transport of carcasses can be eliminated if livestock farms could arrange a way for safely treating this material before being used as feed in the anaerobic digester [126].

Arenas et al. [38] reported a methane yield of 0.47 m^3^ CH_4_/kg VS (volatile solids), when evaluating batch digestion of this material. Biodegradation of proteins can lead to high biogas yields, but the accumulation of ammonia in the digester can cause inhibitory conditions and negatively affect performance. Ammonium ions are released from the anaerobic conversion of proteins inhibiting microbial activity when levels reach values close to 4.0 g/L [127]. Co-digestion of substrates with a low C/N can be alleviated by adding substrates with high carbon content [128]. Thus, a better outcome is expected when adding grass, straws, lignocellulosic biomass or micro-algae biomass as co-substrates.

Fats, oil and grease (FOG) mostly come from food processing industries, slaughterhouses, and food wastes. Usually, this substrate has attracted great attention because of its high methane potential. Compared to the theoretical methane potentials of carbohydrates and proteins −370 and 480 m^3^/kg VS, respectively, FOGs have a value of 1014 m^3^/kg VS [129]. Likewise, co-digestion of FOG with sewage sludge has attracted much attention due to its capability of increasing methane yield by 83% [130]. The counterpart is that co-digestion of FOG may cause problems in digesters such as pipe clogging and foaming that may block gas pipe systems and also cause severe fouling of gas collection pipes [131]. Other studies have reported on digestion problems when FOG is added as co-substrate under the use of short hydraulic retention times [132]. The degradation of FOGs releases long-chain fatty acids, which may hinder the metabolism of methanogenic microflora. Fortunately, these problems may be avoidable by controlling organic loading to the reactor [133], and recently, the use of lipases has proven to be effective in pre-treating fats and increasing degradation performance [104].

## 4. Technical and Economic Feasibility of Anaerobic Digestion Plants

The cost of biogas production by anaerobic digestion presents a wide range of values for each feedstock, mainly due to the economy of scale. It is difficult for biogas to compete with the low prices of natural gas worldwide [134]. Biogas is produced as a mixture of mainly methane and CO_2_, and thus its energy content is much lower. Transport costs and dispersion of substrates are also relevant factors that act as a disadvantage when attempting the valorization of organics. Even though digestate may be considered a valuable agronomic resource, the large quantity produced may become a serious problem when there is not enough land available nearby the digestion plant. If biogas is to be considered as a possible substitute of natural gas or either used as a supplementary fuel in different applications where natural gas is also used, then upgrading is necessary. This increment in costs and the already high installation costs of digestion plants make this mature technology a promising process that finds it very hard to attain economic feasibility. Many digestion plants are unable to survive without the continuous need for fiscal incentives. Still, several advantages can be cited to remark on the benefits of anaerobic digestion. However, the low economic feasibility of these types of plants and the complexity associated with its operation have prevented its further expansion.

The need for pre-treatments to facilitate hydrolysis is usually acknowledged; however, most of these applications are associated with laboratory assays. Industrial plants already have installed pre-treatment equipment to remove inert material, glass and grit, especially when wet-treatment technology is used, to prevent damaging pumps, clogging and avoid extremely high maintenance costs [135]. Dry digestion technologies are capable of managing a greater amount of inert materials, but many of these processes operate under batch conditions and may present lower yields due to the excessive accumulation of ammonia in the lixiviate, particularly if chicken or poultry manure is treated [90,136,137].

Digestion plants have a double function, producing biogas that serves as a renewable fuel to aid in the mitigation of climate change. The second function is the stabilization of organic matter to avoid uncontrollable degradation. However, these two benefits that have a severe impact on the environment still need to cope with the economic factor. The economic success of investing in a digestion plant keeps a close relation with incentive policies adopted by those countries. From a public point of view, the sustainability of energy conversion plants has to be investigated not only considering environmental, energy, and local aspects but also the economic ones [138], because incentives provided to private investors may distort the energy market. It does not seem reasonable to assume that the decarbonization of the economy would be attained through a financial debt that should be paid by conventional energy-producing systems and fossil fuels.

Installation and maintenance costs of digestion plants seem to be still too high. The complex structure of these plants makes operation difficult, particularly those based on wet technology having a lot of piping and pumps. Therefore, the configuration of the equipment in the plant is imperative to reduce distance and prevent difficult clogging situations [135]. To all these facts, the costs of the equipment associated with increasing performance of digestion—hydrolysis units—and upgrading of biogas should be added.

Thermal hydrolysis is the technology that has experienced a widespread on the hand of sewage sludge treatment. The experience gained by the implementation and operation of this technology may serve as a primordial scientific base for adapting these systems and reducing digester size. From the installation of the first full-scale plant for sludge disintegration through Cambi thermal hydrolysis (Hamar, Norway, at the end of 1995) reported by Kepp et al. [11], a lot of knowledge and several designs have been developed to increase the efficiency of the batch thermal hydrolysis process to get fully operational prototypes for continuous processes [139]. To the advantage of smaller digestion units, the lower amount of digestate needing final disposal is added, as well as carrying out the development of new configurations capable of greatly improving dewatering characteristics of digestates [140].

Applied research associated with new technological developments is required to increase biogas production by increasing reactor efficiency and making anaerobic digestion a cost-effective process capable of producing a sustainable fuel with similar characteristics to natural gas [134]. Imeni et al. [141] performed a techno-economic assessment for a digestion plant associated with a livestock farm of 250 adult cattle heads. These authors reported that revenues generated from anaerobic mono-digestion could not offset the initial required investment, thus establishing clear numbers to what is recognized in the industrial digestion sector. Co-digestion is imperative in order to obtain better economic performance and positive returns for farmers willing to implement anaerobic digestion. The other main issues are the price of selling electricity, the price of the co-substrate, transportation distances, and returns from excess heat. Pre-treatments applied to lignocellulosic materials should be carefully evaluated so that the installation of the new equipment associated with the pre-treatment does not exert an extra demand for energy, which may be higher than that obtained from the enhancement in biogas production.

Different economic and technical analyses have been performed, but comparing their results is not an easy task because, as Rajendran and Murthy [142] stated, the lack of standardization of the different assessments makes impossible the direct comparison of results. Other points needing standardization are process mapping, database development, profitability indicators, and regional considerations. Table 2 presents a list of different studies and their assumptions regarding digester size, treatment capacity and revenues. Some of these studies consider a selling price for liquid digestate or the solid fraction of this material, but this assumption may not have real application in some countries. In any case, the main revenues are usually associated with the price of electricity, energy incentives for producing renewable energy, and the price set for treating waste material from other sources. If this later scheme is not possible, then finding suitable co-substrates in the immediacies of the plant may create additional operating costs due to their transport. There are limits based on reasonableness to the distance from where organic wastes are collected to be subsequently treated at a centralized digestion plant [94].

Piñas et al. [143] evaluated the digestion of cattle manure as a single substrate for the Brazilian scenario, reporting that biogas plants presented economic viability for electrical power higher than 740 kWe, and, in the case of co-digestion, the limit was above 1000 kWe. These data indicate that for many small livestock farms, the digestion technology results are completely inadequate. Al-Wahaibi et al. [144] reported good economic performance from the techno-economic evaluation of the digestion of food wastes using a low-cost small digester from a Chinese manufacturer and setting a selling price for biogas of USD 0.22–0.39/m^3^ (equivalent to EUR 0.18–0.32/m^3^ using a factor of USD/EUR 0.82 for currency conversion). Small decentralized digestion may be an option for treating disperse sources of biomasses. However, a smaller scale of the installation would make the in-situ valorization or upgrading of biogas unfeasible. A decentralized biogas plant may be more attractive if the raw feedstock is available near the farm. Thus, transport and storage of co-substrates stop being an issue. Therefore, decentralized biogas plants have clear advantages on islands, farm, and even rural regions [145]. Future studies should focus on combining different scales for optimizing resources and minimizing installation and maintenance costs.

**Table 2:** Assumptions of revenues obtained from digestion plants and capital investment of the installation reported in literature.

Substrates	Selling Prices	Digester	Costs (EUR) Millions	Reference
Dairy cow farm Input: 29,200 t/year Plant treating manure + sheep dung	Electricity: EUR 0.10/kWh Liquid fertilizer: EUR 120/t	2713 m^3^	0.77	[146]
2400 beef cattle and glycerine + biomass as co-substrate	Credit claims. Electricity: EUR 0.012/kWh Solid by-product: EUR 28.8/t Liquid by-product: EUR 2.15/t	2 digesters 3670 m^3^ each	2.55	[147]
8000 t/year Dairy manure, corn stalk, tomato residues	Electricity: EUR 0.13/kWh Heat: EUR 0.0326/kWh Bio-methane: 44/m^3^	1000 m^3^, wet digestion 250 m^3^, solid digestion	0.4–0.5	[148]
Two-phase olive oil mill pomace and pig slurry 7500 t/year and 2450 m^3^/year of wastewaters + pig slurry 9000 t/year	Electricity: EUR 0.13/kWh Heat: EUR 0.036/kWh Waste management savings: EUR 5/t Compost: EUR 70/t Olive stones: EUR 80/t	Plant size to digest 8750 t/year.	0.6–1.1	[149]
Small-scale digestion plants Herd size: 50–250 adult caws)	Electricity: EUR 0.158/kWh Heat: EUR 0.295/kWh District heating selling thermal energy price: EUR 0.03/kWh	Based on installed CHP engine power (kWe): 17–55	0.29–0.52	[150]

Decentralized digestion units may be a suitable solution for treating biomass and organic wastes in regions with a disperse population where land application of digestate and liquid slurry may be possible. Biogas produced from these different systems may be transported and collected in a central up-grading plant or used for electricity production. This approach takes advantage of the fact that levelized costs of electricity are lower for large-scale plants due to the use of more efficient conversion devices and their lower capital cost per unit of electricity produced [151]. It also presents the advantage of treating biomass and wastes locally where they are produced.

**Figure 1:**
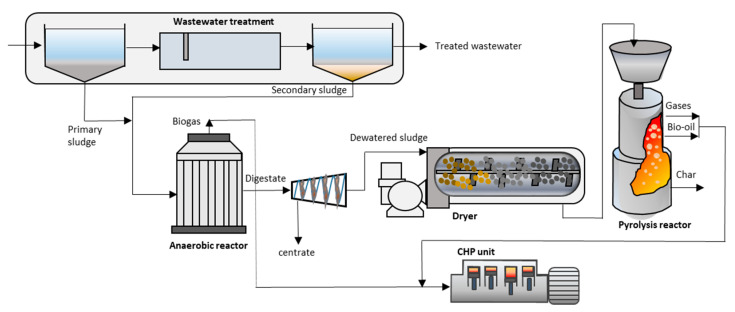
Schematic representation of a wastewater treatment plant (WWTP) converted into an energy producing center. Anaerobic digestion and pyrolysis are integrated for the combined treatment of sewage sludge.

Increasing the efficiency of digestion processes to optimize the production of energy has been attempted by integrating thermal conversion for treating the solid fraction of digestates or treating the non-organic fraction of the feeding stream [152,153,154,155]. This is particularly adequate for centralized treatment plants with no possibility of land digestate disposal. The evaluation of performance under different configurations and types of organic materials has been studied by González-Arias et al. [156,157] and Ghysels et al. [158] analyzing the global process performance of digestate pyrolysis and co-pyrolysis with lignocellulosic biomass. Life cycle analysis has also been applied to the integrated approach of digestion and pyrolysis as a treatment option for waste management [159], indicating a higher conversion rate of the raw material to energy [160]. In fact, current wastewater treatment plants (WWTPs), where anaerobic digestion is widely applied, can be transformed into a treatment center for energy production. The dried anaerobic sludge can be converted into biochar, bio-oil, and bio-syngas using a pyrolysis reactor. The energy contained in bio-syngas and bio-oil can be exploited using a CHP system or a dual combustion system, and biochar can be used as a soil additive in agriculture [161]. Figure 1 represents a schematic configuration of this approach.

These proposals that may seem technically feasible still have to overcome excessive installation costs of pyrolysis units. Trippe et al. [162] evaluated the techno-economic analysis of a pyrolysis installation for a plant with a capacity of 100 MW thermal energy input indicating a price between EUR 40.5 and 47 million. Campbell et al. [163] estimated a value of USD 76.7 million as total capital investment for a pyrolysis plant capable of handling 65.7 kt dry feedstock annually. Shahbaz et al. [164] estimated a cost of USD 9 million for a plant with a treatment capacity of 438 t/year of lignocellulosic material. Whatever the assumption made to carry out the economic evaluation, the profitability of these plants may be completely out of question. One of the factors that greatly affects the economic feasibility of these plants is the costs associated with catalysts, which can contribute on average about 15% of the total fuel cost, when upgrading this liquid fuel is intended to obtain a stable oil product with low oxidation tendency. The yield of bio-oil is also a relevant parameter affecting the process economics [165]. However, getting back into the valorization of biomass, the management based on the combined performance of the two processes, that are digestion and pyrolysis, is a matter that at least, at the current state of the art, is not possible due to the excessive costs of capital investment of these units. Different parameters may be considered as relevant when evaluating any of these processes. In any case, the initial investment is so high that it hardly can become a reasonable solution to any livestock farm for treating manures.

The recent pandemic caused by the novel coronavirus (SARS-CoV2) has affected several countries regardless of the economic level and it has set a lot of pressure on governments to focus on protecting lives. The pandemic also created an enormous challenge for the health care sector and completely changed the dynamic of waste generation, causing a steep increase in the amount of medical hazardous wastes and plastics associated with measurement for preventing infections [166]. There has been an increase in the demand for plastic-packaged food and groceries, and the use of disposable utensils. The current use of disposable face masks has increased, and there is excessive use of gloves recommended in supermarkets during the early spring when the negative effects of the pandemic hit developed countries. The use of single-dose packaging also increased along with single-use plastics as a way for implementing hygiene measurements. These new customs greatly increased the use of plastic materials and thus the amount of wastes produced [167]. Any waste management systems already presenting inefficiencies have probably experienced an aggravated situation due to the increase in the volume of plastics, which in turn may be triggering a new environmental crisis [168].

Given the high costs for operating waste treatment plants and the number of resources necessary to deal with health measurements, it is probable that investments in clean energy systems will be delayed or even cancelled. Although there is great uncertainty about the future of many industrial sectors and the fate of the economy, it is reasonable to assume that due to the COVID-19 outbreak, the renewable energy sector may be negatively affected because of the need for placing energy incentives to a second place due to the large number of incentives put into practice by countries in the fight against the COVID-19 outbreak [169]. Therefore, it is imperative to find ways to effectively manage waste treatment plants and produce renewable energy at a reasonable cost to accomplish climate change goals and keep the economy running in line with sustainability principles.

Nagaj and Korpysa [170] studied the effect of the COVID-19 pandemic on the level of energy poverty in Poland, reporting a negative impact on the increase in prices and expenditure on energy carriers, with the most affected being the poorest households. These results may probably have an extrapolation to other European and non-European countries. Therefore, the arguments generally accepted of considering whether it is fair to pay higher prices for electricity and heat produced from renewable sources when the economy is rolling into a crisis scenario will no longer be acceptable since it will only set more pressure on households that are already experiencing financial problems.

## 5. Integrating Anaerobic Digestion into a Green Energy Producing System

The great capacity of anaerobic digestion to treat a wide variety of substrates and stabilize organic compounds has made this technology a useful ally in the development of the biorefinery concept for producing green products. The transformation processes involved in this approach are those specifically designed and optimized for treating renewable raw materials. As defined by Clark and Deswarte [171], a biorefinery is a facility capable of converting biomass, including waste materials, into a variety of chemicals, biomaterials and energy, maximizing in these transformations the value of biomass and minimizing the production of final wastes. A biorefinery may be analogous to today’s petroleum-based refineries with the difference that renewable biomass is the main resource used as feedstock [172].

There are several studies found in the literature proposing the valorization of biomass using the biorefinery concept to obtain different products and energy [173,174,175,176]. It is necessary to evaluate the energy demand, installation costs, operating and maintenance costs of the different concatenations of processes dedicated to attaining full valorization of biomass and wastes. In the case of valorizing lignocellulosic biomass, hydrolysis is an essential step, and therefore, the development of continuous operating reactors for the fractionation of biomass is crucial to increase product yield [177]. Batch processes are well developed and several years of experience have been gained in the operation of these units. The future development of thermal hydrolysis of lignocellulosic biomass under continuous operation should be closely related to those designs that are currently being tested under industrial large scale for continuous thermal pre-treatment of sludge [12,178] operating at high solid content as the Cambi Solidstream^®^ (Cambi, Asker, Norway) process [179].

The different processes associated with the conversion of biomass, lignocellulosic biomass or organic waste materials are usually characterized by having a high energy demand, usually needing high temperature and pressure for performing hydrolysis, or in the case of some fermentation processes, these may need a costly cleaning step, aeration and some even sterilization. Developing and evaluating new methods for reducing energy consumption is of great relevance. An example of the different fields where energy efficiency can be improved is the technological development of Ren et al. [180], who proposed a novel isothermal compression method to lower energy consumption by designing an isothermal piston where a porous medium was placed. The design allows one to absorb the compression heat, and this heat is then conducted through the liquid at the chamber bottom. This way, the heat transfer can be significantly enhanced, due to the large surface area of the porous medium. As the liquid has a large heat capacity, its temperature can be kept constant through circulation, creating near-isothermal compression and minimizing energy loss in the form of heat, which cannot be recovered. These findings are crucial because attaining higher efficiencies directly impacts the economic feasibility of plants. If decarbonization of the economy is a primary goal, and it is a goal to be kept even under a pandemic scenario with an imminent economic crisis being expected, the environmental measurements to be taken and the transition to a cleaner energy production system should be performed without the need for excessive fiscal incentives.

Biomass is considered an inexpensive feedstock with a great potential to replace a wide diversity of fossil-based products within the energy sector; heat, power, fuels, materials and chemicals, but the effective utilization of biomass constituents is necessary. The conversion of biomass can be classified into five major steps: choice of suitable biomass, effective pre-treatment, production of saccharolytic enzymes-cellulases and hemicellulases, fermentation of hexoses and pentoses and downstream processing [181]. Techno-economical assessments are necessary to develop a suitable transformation process. Biorefineries have also been classified based on the characteristics of the different transformation processes involved by Fernando et al. [182]. These authors proposed three phases for classifying biorefineries depending on the flexibility of input, processing capabilities, and product generation. Phase I is those having less or no flexibility in any of the three aforementioned categories. Phase II allows flexibility in product generation while having fixed input and processing capabilities. Phase III is biorefineries presenting flexibility in all the three previous categories and can be defined using the concept of high-value low-volume (HVLV) and low-value high-volume (LVHV) outputs.

In any processing chain, the production of by-products of low value is always inevitable. At this point, other processes can be introduced as a way of improving global performance, but this should be done without excessively increasing capital investment costs or the energy demand. Table 3 shows different substrates being processed for obtaining different valued products under the biorefinery concept. The number of publications evaluating this type of approach is enormous, thus indicating the great interest in the scientific community for making these approaches a reality. Table 3 also shows a list of some publications considering the coupling of different treatment processes along with anaerobic digestion.

Alves et al. [183] performed a techno-economic assessment under the Brazilian scenario to produce renewable jet fuel. These authors studied the use of different biomass feedstock (sugar crops, oil crops, and lignocellulosic biomass) for producing biojet fuel and higher value-added products in a biorefinery platform. As a result, they demonstrated that the main specific factors that should be accounted for to make this approach a real technical solution were, of course, the cost of feedstock and the selling price of biochemicals. The investment costs considered by these authors for the biorefinery installation were of the order of USD 75–800 M for the biojet producing section when analyzing different conversion technologies and of USD 10–278 M for chemical products. Under a high uncertainty scenario, as is the current one, it is not easy to achieve such type of projects. Still, lessons are to be learned from the different proposals, given that the extensive literature is now available regarding the possible processing routes for biomass conversion.

**Table 3:** Biorefinery: substrates and products obtained from this approach. Anaerobic digestion integrated into this concept. Data obtained from literature reports.

Substrates	Conversion Processes	Products	Reference
Jerusalem artichoke (*Helianthus tuberosus* L.)	Extraction	Sugars, succinic acid, Rubisco fraction, proteins	[172]
Invasive brown algae (*Sargassum muticum*)	Drying, extraction, fractionation	Fucoxanthin and hydrolysis liqueur	[184]
Food waste	Black soldier fly (BSF) (*Hermetia illucen*)	High-value insect products: protein, lipids, chitin and frass	[185]
Sugar beet	Ethanol fermentation + continuous fermentation with *Bacillus coagulans*	Ethanol and lactic acid	[186]
Grape wine waste	Extraction + hydrolysis + fermentation	Lactic acid, tartaric acid, protein-rich fungal biomass, tannins, polyphenols	[187]
Sugarcane distillery (bagasse) and straw	Fermentation + gasification + Fischer–Tropsch synthesis	Ethanol, diesel, jet fuel, gasoline, electricity	[188]
Citrus wastes	Pectin extraction anaerobic digestion	Mucic acid production and biogas	[189]
Agricultural and livestock wastes	Fermentation + anaerobic digestion	Biogas, electricity, ethanol, butanol, acetate, propionic, lactic acid	[4]
Switchgrass (*Panicum virgatum*)	Ethanol fermentation + anaerobic digestion + extraction + thermal process	Ethanol, biogas, electricity, phenol	[190]
Sugarcane bagasse (*Saccharum officinarum*)	Enzymatic hydrolysis, fermentation + anaerobic digestion + combustion	Ethanol, biogas, heat	[191]
Meat processing wastes	Hydrolysis − esterification + anaerobic digestion + hydrothermal liquefaction	Biodiesel, biogas, biochar, bio-oil	[192]
Wheat straw Animal bedding	Pre-treatment + hydrolysis + fermentation + anaerobic digestion	Ethanol, biogas, energy	[193]

**Figure 2:**
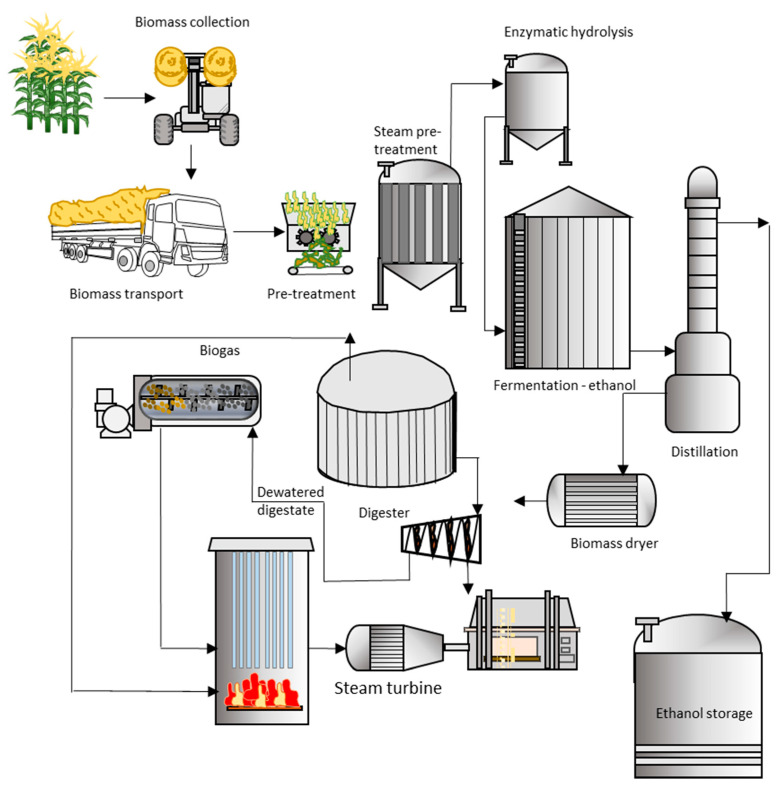
Schematic representation of the different processes involved in a biorefinery to transform biomass into chemicals and energy.

Figure 2 shows a schematic representation of the different products and processes involved in a biorefinery to transform a variety of available biomasses. Key issues considered in this scheme are biomass selection, transport costs, high and low-value products, conversion yields, energy efficiency and energy recovery. Other relevant factors when evaluating the feasibility of biorefineries are those considered by Zetterholm et al. [194]. The framework considered by these authors included the competition for biomass across sectors, also assuming exogenous end-use product demand, incorporating various geographical and technical constraints. Their study considered a sawmill-integrated biorefinery producing liquefied biomethane from forestry and forest industry residues. Their study illustrates the relevance in acknowledging biomass market effects in the supply chain and, therefore, economic performance. Technologies selected when dealing with the processing of biomass should be capable of adjusting to changes in feedstock prices. A biorefinery is a high capital investment facility and, as stated by Zetterholm et al. [194], these types of installations have many relevant decision variables; ignoring some of their key aspects may result in misleading conclusions and conflicting policy recommendations.

## 6. Conclusions

A review regarding the main advantage of anaerobic digestion is presented in this manuscript; it considers this process essential for biomass valorization and focuses on the benefits that this process offers in producing biofuels and energy recovery. Increasing the efficiency of the global processing chain and decreasing installation costs are primordial if decarbonization of the economy is to be taken as a goal to achieve in the recent future. Bio-energy production and cleaner energy systems need to be developed following the principles of sustainability and circular economy, but these premises also imply attaining economic feasibility without depending on subsidies from incomes that in turn are derived from fossil energy-producing activities.

Digestion is a technology expected to play a major role in producing renewable energy and climate change mitigation, but economic feasibility needs to be addressed. The excessive capital investment of these installations is unaffordable for many small livestock farmers; therefore, solutions need to be found considering local characteristics and possible additional revenues from the production of chemical products in addition to energy.

The future outlook of this technology is closely linked to its capacity to transform a wide variety of organics under high loading conditions; therefore, this technology should be considered as an important player when developing future production systems to attain higher efficiencies in the use of energy and resources. The integration of anaerobic digestion with different processes with the aim of further developing the biorefinery concept is an alternative that deserves a deeper evaluation. Biorefineries can easily connect with the local population and reactivate many regions suffering from depopulation due to the lack of working opportunities. Thus, anaerobic digestion, understood as a renewable energy production process, may open the door to the development of a completely new sustainable society. The main parameters to be carefully evaluated to achieve economic feasibility should consider transport costs, substrate treatment flexibility, price and market distortions and seasonality, energy demand and maintenance costs associated with pre-treatment units, selling prices of goods and treatment costs of side-streams.
